# A Transcriptome for the Study of Early Processes of Retinal Regeneration in the Adult Newt, *Cynops pyrrhogaster*

**DOI:** 10.1371/journal.pone.0109831

**Published:** 2014-10-07

**Authors:** Kenta Nakamura, Md. Rafiqul Islam, Miyako Takayanagi, Hirofumi Yasumuro, Wataru Inami, Ailidana Kunahong, Roman M. Casco-Robles, Fubito Toyama, Chikafumi Chiba

**Affiliations:** 1 Graduate School of Life and Environmental Sciences, University of Tsukuba, Tsukuba, Ibaraki, Japan; 2 Faculty of Life and Environmental Sciences, University of Tsukuba, Tsukuba, Ibaraki, Japan; 3 Graduate School of Engineering, Utsunomiya University, Utsunomiya, Tochigi, Japan; University of Dayton, United States of America

## Abstract

Retinal regeneration in the adult newt is a useful system to uncover essential mechanisms underlying the regeneration of body parts of this animal as well as to find clues to treat retinal disorders such as *proliferative vitreoretinopathy*. Here, to facilitate the study of early processes of retinal regeneration, we provide a *de novo* assembly transcriptome and inferred proteome of the Japanese fire bellied newt (*Cynops pyrrhogaster*), which was obtained from eyeball samples of day 0–14 after surgical removal of the lens and neural retina. This transcriptome (237,120 *in silico* transcripts) contains most information of cDNAs/ESTs which has been reported in newts (*C. pyrrhogaster*, *Pleurodeles waltl* and *Notophthalmus viridescence*) thus far. On the other hand, *de novo* assembly transcriptomes reported lately for *N. viridescence* only covered 16–31% of this transcriptome, suggesting that most constituents of this transcriptome are specific to the regenerating eye tissues of *C. pyrrhogaster*. A total of 87,102 *in silico* transcripts of this transcriptome were functionally annotated. Coding sequence prediction in combination with functional annotation revealed that 76,968 *in silico* transcripts encode protein/peptides recorded in public databases so far, whereas 17,316 might be unique. qPCR and Sanger sequencing demonstrated that this transcriptome contains much information pertaining to genes that are regulated in association with cell reprogramming, cell-cycle re-entry/proliferation, and tissue patterning in an early phase of retinal regeneration. This data also provides important insight for further investigations addressing cellular mechanisms and molecular networks underlying retinal regeneration as well as differences between retinal regeneration and disorders. This transcriptome can be applied to ensuing comprehensive gene screening steps, providing candidate genes, regardless of whether annotated or unique, to uncover essential mechanisms underlying early processes of retinal regeneration.

## Introduction

The newt has long been recognized as a master of regeneration from which the principles for the regeneration of body parts following traumatic injury can be learnt. This animal has an outstanding ability, when metamorphosed or sexually matured, or even as an aged adult, to regenerate missing body parts – including a part of a limb, the jaw, the tail (the spinal cord), the brain, the heart, the eye (the lens and the retina) – from remaining tissues at the site of injury. This takes place through dedifferentiation–redifferentiation/-transdifferentiation (or reprogramming) of terminally differentiated cells as well as recruitment of endogenous stem/progenitor cells, their proliferation and patterning, and physiological integration of regenerates into the body system [1], [2]. Although ample endeavors have been made to understand these surprising phenomena for over a century, the underlying cellular mechanisms and molecular networks are still largely uncertain primarily because of our technical limitations. However, studies in this field are now moving forward with increasing speed by incorporating highly efficient technologies to analyze gene functions [3], [4] and transcriptomes [5], [6]. For studies of the newt whose genomic size is too large to be sequenced (estimated ∼20 Gbp [7]), construction of an *in silico* transcriptome by RNAseq/*de novo* assembly is certainly cost effective, and the resulting data sets are highly informative for bench screening candidate genes.

Regeneration of the adult newt retina is a good system to study the early processes of regeneration, particularly the mechanisms underlying reprogramming, cell-cycle re-entry/proliferation and patterning, because of its simplicity. The cell source for retinal regeneration in the posterior eye is the retinal pigment epithelium (RPE) cells only. The RPE, which is a highly specialized monolayer lining the back of the neural retina, expresses specific molecular markers such as RPE65, allowing the RPE cells and RPE-derived cells to be tracked in an early phase of retinal regeneration [8]. In addition, this system has an obvious medical target, namely *proliferative vitreoretinopathy* (*PVR*) in which RPE cells proliferate and transform in response to retinal injury, leading to the loss of vision [9].

Among all newt species, the Japanese fire bellied newt (*Cynops pyrrhogaster*) is the best choice for the study of retinal regeneration since: 1) a surgical procedure to induce retinal regeneration has been established; 2) morphological stages of retinal regeneration have been defined in detail; 3) both *in vivo* and *in vitro* functional gene assay systems are being developed [4], [8], [10], [11]. However, to facilitate the study of retinal regeneration in this species, one considerable obstacle still remains: limited information on genes. Thus, in the current study, to overcome this problem we carried out mRNA-seq/*de novo* assembly in this species, providing an *in silico* reference transcriptome specialized for the study of early processes of retinal regeneration.

## Materials and Methods

### Ethics statement

This study using the Japanese fire bellied newt *C. pyrrhogaster* was permitted by the University of Tsukuba Animal Use and Care Committee (AUCC). Surgical removal of the neural retina (retinectomy) and sacrifice were carried out under anesthesia [anesthetic: FA100 (4-allyl-2-methoxyphenol); DS Pharma Animal Health, Osaka, Japan] to minimize suffering [8]. No other *in vivo* experiments were done.

The field study did not involve endangered or protected species. The newts were originally captured by a provider (Mr. Kazuo Ohuchi, Misato, Saitama, 341-0037 Japan; http://homepage3.nifty.com/monmo51-kaeru/index.html) using a net from canals along the rice paddies located within ∼25 km in diameter around a Miyayama area (35.130013,140.013842) in Kamogawa city, Chiba prefecture, Japan [4]. No specific permissions were required for the location of capture.

### Newt strain

The newts, which have been captured since 2008 (∼300/year) (see Ethics statement), have been stocked/cultured in both the laboratory (Univ. of Tsukuba) and a field ‘Imori-no-Sato’ (Kaizuka/Kamitakai, Toride city, Ibaraki prefecture, Japan; http://imori-net.org/) [4]. This population belongs to Kanto group in Northern lineage [12] and is called ‘Toride-Imori’. In this study, sexually mature adult Toride-Imori (total body length: male, ∼9 cm; female, 11–12 cm) which had been reared in the laboratory were used.

### Housing condition

In the laboratory, the animals had been reared in containers/aquarium tanks (≤1 newt/base area of 50 cm^2^ square; the water depth was 5–15 cm; a stone/a piece of kitchen sponge was placed therein, serving as land) at ∼18°C under a natural light condition; they had been fed with frozen mosquito larvae (Akamushi; Kyorin Co., Ltd., Japan) every day and the containers/aquarium tanks had been kept clean [4].

### Anesthesia

For retinectomy, the animals were anesthetized as follows: 0.3 ml of the anesthetic FA100 (4-allyl-2-methoxyphenol) was poured in 300 ml tap water (∼22°C) in a bottle (φ of the bottom: ∼7 cm; height: ∼5 cm) with a lid; the bottle was sealed immediately and shaken several times so that the solution is mixed well; the newts (up to 5) were placed in this solution (i.e., 0.1% FA100) and the bottle was sealed again immediately; the bottle containing the animals was placed in the dark at room temperature (∼22°C) for 2 h, allowing dark adaptation of the retina which makes the adherence between the neural retina and the RPE weaker. After this treatment, they were rinsed in distilled water (DW) and wiped with a paper towel. Under this condition, they did not show the pupillary reflex during surgery, and not awake for at least 4 h.

For sacrifice, intact animals and those of day-14 or later after retinectomy whose wound has closed were anesthetized as done for retinectomy. However, in the case of animals between 4 h and day-14 after retinectomy, an alternative anesthetic method was applied to avoid damage of RPE cells due to invasion of the hypotonic anesthetic solution into the eye chamber: the animals were injected with 100 µl of 20% FA100 (dissolved in PBS) into their abdominal cavity through a fine needle (27Gx3/4″, NN-2719S, Terumo, Tokyo, Japan) connected to a syringe (1 ml, SS-01T, Terumo); they fell asleep in 30 min. For animals within 4 h after retinectomy, additional anesthetic treatment was not done.

### Retinectomy

**Figure 1:**
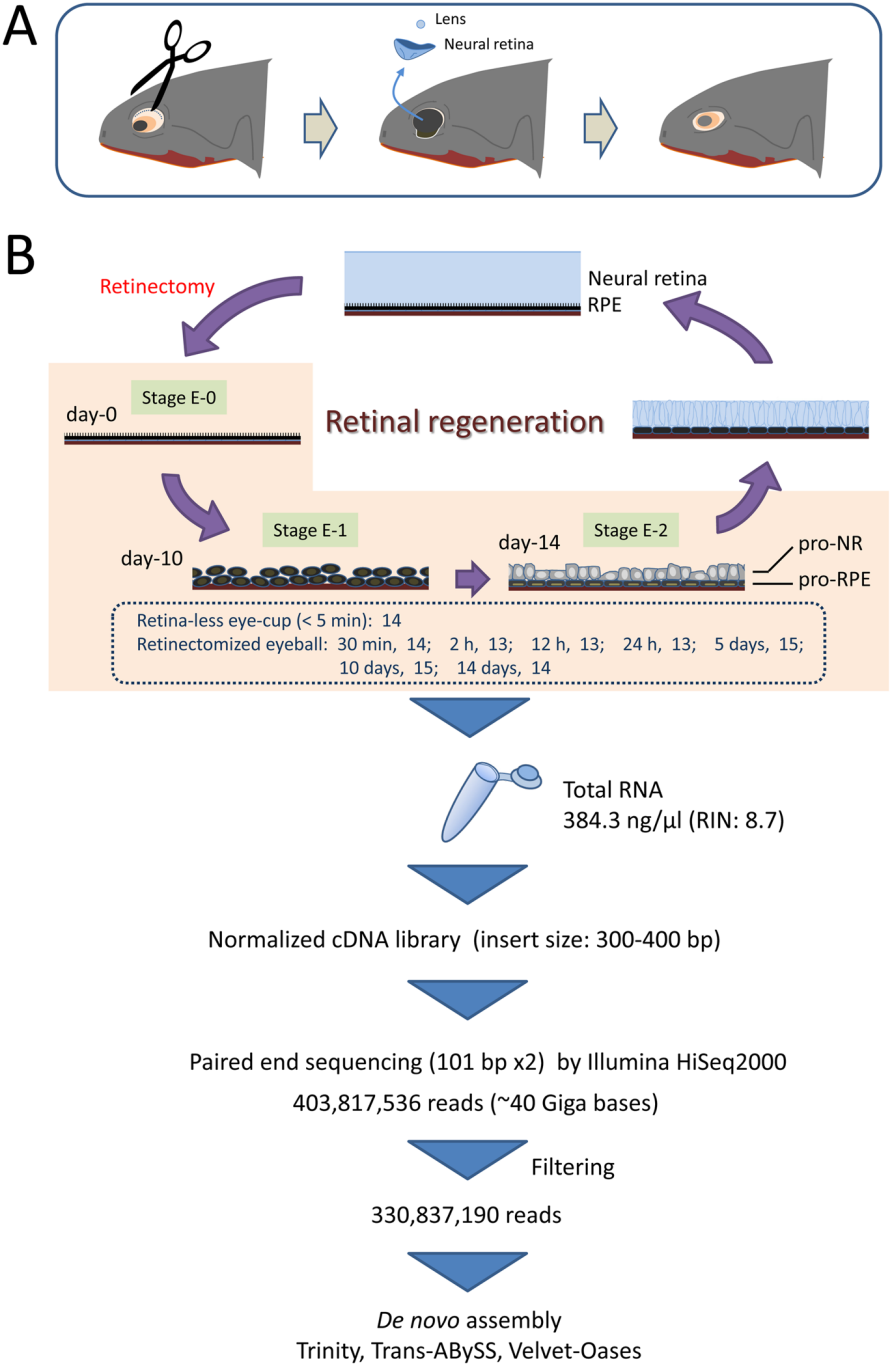
Workflow from retinectomy to *de novo* assembly. **A.** Retinectomy. **B.** Sample collection, mRNA-seq and *de novo* assembly. *Stage E-0*: The RPE immediately after retinectomy. *Stage E-1*: Almost all RPE cells that have lost their epithelial characteristics and formed aggregates have entered the S-phase of the cell-cycle. *Stage E-2*: Partially depigmented cells are segregated into two rudiment layers (pro-NR and pro-RPE), which give rise to a new neural retina and the RPE layer itself. Under the current experimental conditions, regenerating retinas at Stage E-1 and E-2 are obtained at day-10 and -14 po [8].

To induce retinal regeneration, the neural retina was removed, together with the lens, from the left eye (the eyeball size: 2 mm in diameter) of a living animal as follows ([8]; see Figure 1A): the mouth cavity of an anesthetized animal was stuffed with a roll of the absorbent cotton so that the eyeball is pushed out from the eyelid; the animal was held on the silicon bottom of an operating chamber so that the left eye faces up, using a U-shaped pin which was mounted on the neck of the animal and stuck onto the silicon bottom of the chamber; the animal on the chamber was placed under a binocular, and the dorsal half of the left eye was cut open along the position slightly below the boundary between the cornea and sclera using a blade and fine scissors; both the neural retina and the lens were carefully removed by a fine injection needle (27Gx3/4″, NN-2719S, Terumo) and forceps, while gently infusing a sterile saline solution (in mM: NaCl, 115; KCl, 3.7; CaCl_2_, 3; MgCl_2_, 1; D-glucose, 18; HEPES, 5; pH 7.5 adjusted with 0.3N NaOH) into the vitreous chamber through the same injection needle which was connected to a syringe (1 ml, SS-01T, Terumo) via a filter cassette (0.20 µm pore size, Cellulose acetate, DISMIC-25CS, ADVANTEC, Japan); at this time the retinal margin containing the *ora serrata* (the tissue harboring the retinal stem/progenitor cells) which remained along the base of the ciliary epithelium was also removed by forceps. After operation, the eye flap consisting of the iris and cornea was carefully placed back to its original position. The operated animals were placed on a paper towel (lightly wet with DW) in a plastic container [≤5 newts/container (width: 14 cm×depth: 19 cm×height: 4 cm)] and allowed to recover, and then reared in an incubator (∼22°C; the day-night cycle was 12 h:12 h) until use (up to 14 days in this study). In the mean time, the containers were kept clean and the animals were not fed to control the speed of regeneration. The stage of retinal regeneration and corresponding day post-operation (po) were determined according to previous criteria ([8]; see Figure 1B).

### Collection of eyeballs

To collect eyeballs, the anesthetized animals (dried on a paper towel) were decapitated. The head was fixed on the silicon bottom of the operating chamber with a marking pin, and placed under the binocular. The eyeballs were carefully enucleated with fine scissors and forceps. In the case of retinectomized animals, especially before day-14 po, this operation was made with minute attention because their wounded eyes were very fragile.

### Tissue samples for analyses

For *de nove* assembly transcriptome, the workflow is illustrated in Figure 1B. To obtain the transcriptome involved in early processes of retinal regeneration, especially for the study of reprogramming, cell-cycle re-entry/proliferation and patterning in full, retinectomized eyeballs (time po: 30 min, 2 h, 12 h, 24 h, 5 days, 10 days, 14 days) and the retina-less eye-cups (RLECs) of normal eyeballs were used. These retinectomized eyeballs should have contained RPE cells which just received somewhat onset-signals for regeneration [13], [11] as well as those which have undergone mitosis (stage E-1) and segregation into two layers, i.e., rudiments for a new neural retina and RPE (stage E-2) ([8]; see Figure 1B for the morphological stages of retinal regeneration). RLECs were used as a source of intact RPE cells (stage E-0). This sample was prepared as described previously [8] with some modifications: a normal eyeball was placed, the cornea side up, onto a filter membrane (MF™ Membrane Filter, 0.45 µm HA, HAWP01300, Merck Millipore, Darmstadt, Germany) in a 35 mm plastic dish (Falcon 353001, Becton Dickinson, NJ 07417-1886), and then cut along the equator by manipulating a blade and scissors; RNase-free PBS chilled on ice was gently poured onto the eyeball; after the dish was filled up with this saline, the anterior half of the eyeball containing the iris and lens was removed to make the eye-cup; the neural retina was carefully separated from the RPE with a fine pin and forceps, and then removed from the rest of the eye-cup, i.e., the RLEC, by cutting the optic nerve with fine scissors. This operation was completed within 5 min.

For quantitative PCR (qPCR), both the right (intact) and left (retinectomized) eyeballs of animals at day-10 and -14 po were used; that is, the right eyeballs were used for day-0 po sample containing intact RPE (stage E-0), and the left ones were for samples of day-10 po (stage E-1) or day-14 po (stage E-2). Tissue samples for RNA isolation were prepared as follows. After the animal was decapitated under anesthesia, the right and left eyeballs were collected in different dishes (filled with RNase-free PBS) on ice. The right eyeball was immediately dissected into the RLEC, and the RPE sheet together with the choroid tissues was isolated by separating these from the sclera using a fine pin and forceps. On the other hand, the left eyeball of day-10 or -14 po, which was put on the filter membrane and soaked in chilled RNase-free PBS as done for the right eyeball, was carefully opened from the wound at the time of retinectomy using a fine pin and scissors; after the anterior part of the eyeball containing the iris and the ciliary marginal zone was carefully removed, RPE -derived cells in the posterior eye were collected together with the choroid tissues as done for the right eyeball. After blood cells in the choroid were removed as much as possible by shaking them in the dish, each day samples were transferred into different tubes (containing RNase-free PBS) on ice with a pipette (3.5 ml Transferpipette, Sarsted, D-51588 Nümbrecht, Germany). For one round of RNA purification, this process was repeated for at least 5 animals, collecting 5 each-day samples (good samples only) at once.

### Library construction and sequencing for *de novo* assembly transcriptome

A total of 14 RLECs and 97 retinectomized eyeballs (30 min, 14; 2 h, 13; 12 h, 13; 24 h, 13; 5 days, 15; 10 days, 15; 14 days, 14) were harvested, under a conventional nuclease free condition, one by one in a 50 ml tube containing liquid nitrogen, and stored in –80°C until use (see the workflow in Figure 1B). Total RNA was purified from them all by the Isogen protocol (Nippon Gene, Tokyo, Japan) according to the manufacturer’s instructions and evaluated with an Agilent 2100 Bioanalyzer (Agilent Technology, Santa Clara, CA 95051). Using a qualified RNA sample (384.3 ng/µl, RIN: 8.7), a normalized cDNA library (insert size: 300–400 bp) was constructed with a TrueSeq RNA Sample Prep Kit (Illumina, San Diego, CA 92122) followed by Duplex Specific Nuclease normalization (Illumina) according to the manufacturer’s instructions. Subsequently, paired end sequencing (101 bp read×2) was carried out by Illumina HiSeq2000 and a set of raw read data [403,817,536 reads; 40,786 Mbases; % of high quality base (≥Q30): 92.74; Mean quality score: 35.92] was produced after base calling and Chastity filtering (CASAVA ver.1.8.1., Illumina). These raw reads were filtered to remove reads with adaptors and low-quality sequences (reads with unknown sequences ‘N’) as follows: first, the software cutadapt (version 1.0; http://code.google.com/p/cutadapt/) [14] was used to trim adapters, and then the trimmed reads and reads containing ‘N’ are discarded using in-house scripts. Finally, 330,837,190 clean reads (101 bp each) were yielded for the following *de novo* assembly.

### *De novo* assembly

To obtain contigs and *in silico* transcripts (*IS*-transcripts), the clean reads, obtained after filtering the raw reads, were assembled using *de novo* assemblers. There is no consensus in terms of the best *de novo* assemblers. So, three widely used algorisms were applied: Trinity [15] (version 2012-10-05; http://trinityrnaseq.sourceforge.net/), Trans-ABySS [16] [for Trans-Abyss, version 1.4.4 (http://www.bcgsc.ca/platform/bioinfo/software/trans-abyss); for ABySS [17], version 1.3.5 (http://www.bcgsc.ca/platform/bioinfo/software/abyss)] and Velvet-Oases [18] [for Velvet [19], version 1.2.01 (http://www.ebi.ac.uk/~zerbino/velvet/); for Oases, version 0.2.02 (http://www.ebi.ac.uk/~zerbino/oases/)]. Trinity was run with ‘–min_kmer_cov 2’. By applying the ‘–min_kmer_cov 2’ parameter (the default is 1), only k-mers that occur more than once are assembled. This parameter is used to reduce memory requirements and runtimes. Generally, this will eliminate super rare transcripts and sequencing errors, but will not usually affect assembly quality. ABySS was run with k-mer values form 51 to 95 in steps of 2, and then all 23 assemblies from AbySS were merged into one assembly using Trans-ABySS. Velvet was run with k-mer values from 45 to 95 in steps of 5, and then the contigs produced by Velvet at each k-mer value were further assembled into transcripts using Oases. Finally, the best result (k-mer = 75) was selected as output. Although the transcript data sets assembled at different k-mer values were generally merged using Oases-M, we just treated Velvet-Oases as a single k-mer assembler without Oases-M.

In this study, *IS*-transcripts deduced by Trinity were further analyzed by annotation as well as coding sequence (CDS) and protein/peptide prediction.

### Annotation

Functional annotation of *IS*-transcripts was carried out by aligning them first to protein databases such as NCBI NR (release-20130408), Swiss-Prot (release-2013_03), KEGG (release 63.0) and COG (release-20090331) by blastx program (E-value threshold: e-5), and then to NCBI NT by blastn program (E-value threshold: e-5). Gene Ontology (GO) annotation was carried out by the Blast2GO program (v2.50) with NR annotation, and the data was classified by WEGO software [20]. Metabolic pathway analysis was carried out with the help of the KEGG database.

### Coding sequence (CDS) and protein/peptide prediction

Both the nucleotide sequence (5′-3′) and protein/peptide CDS of *IS*-transcripts were predicted using proteins with highest ranks in the functional annotation by blastx program with NR, Swiss-Prot, KEGG and COG (see above). *IS*-transcripts that could not be aligned to any databases were scanned by ESTScan (v3.0.2) to predict CDSs [21]. This program compensates for the frame shift errors.

### Quantitative PCR (qPCR)

Each day samples (5 samples/tube), which were prepared and harvested under a conventional nuclease free condition (see above**)**, were immediately used to purify total RNA (NucleoSpin RNA kit; Macherey-Nagel GmbH & Co. KG, Düren, Germany). First strand cDNAs were synthesized (SuperScript™ II Reverse Transcriptase; Life Technologies, Carlsbad, CA) with 30 ng total RNA, diluted 100x, and then used as a template for qPCR. qPCR was performed by a LightCycler Nano system (Roche Applied Science, Penzberg, Germany) according to the manufacturer’s instructions of FastStart Essential DNA Green Master (Roche) or FastStart Essential DNA Probes Master (Roche), with 45 cycles. ID numbers of target *IS*-transcripts (*Ef1α*, *RPE65*, *CRALBP/RLBP1*, *ZO1*, *Otx2*, *Musashi1a/c*, *Cyclin D1*, *CDK4*, *Histone H3*, *c-Myc*, *Klf4*, *Sox2*, *N-Cadherin*, *α-SMA*, *Vimentin*, *Pax6*, *Chx10/Vsx2*, *FGFR1*, *FGFR3*, *Mitf*, *Wnt2b*), and their PCR primers and probes [selected from the Roche Universal Probe Library (https://www.roche-applied-science.com)] are listed in Table S1. The DDBJ/GenBank accession number of the cDNA corresponding to each *IS*-transcript is also mentioned in Table S1. Most cDNAs were cloned and their sequences, determined by the Sanger method, were deposited in DDBJ.

For each gene, qPCR, which was always run simultaneously with day-0, -10 and -14 po samples, was repeated using more than three sets of independently collected samples. The relative expression level of each transcript was calculated as follows: the amount of transcript was first compensated for *Ef1α* (the mean from several rounds of qPCR) in the same sample, and then normalized against that of the day at which the average value from all samples was highest. The data were presented as the mean ± SEM. Statistical differences were evaluated by Sheffe’s test following the Friedman test.

### Data availability

All relevant raw data have been deposited to NCBI [accession numbers: Sequence Read Archive ID, SRP034152; BioProject ID, PRJNA231688; BioSample ID, SRS515156; Accession; SRX391946; Run, SRR1051839] and IMORI [URL: http://antler.is.utsunomiya-u.ac.jp/imori/].

## Results and Discussion

### Three transcriptomes deduced by different *de novo* assemblers

**Figure 2:**
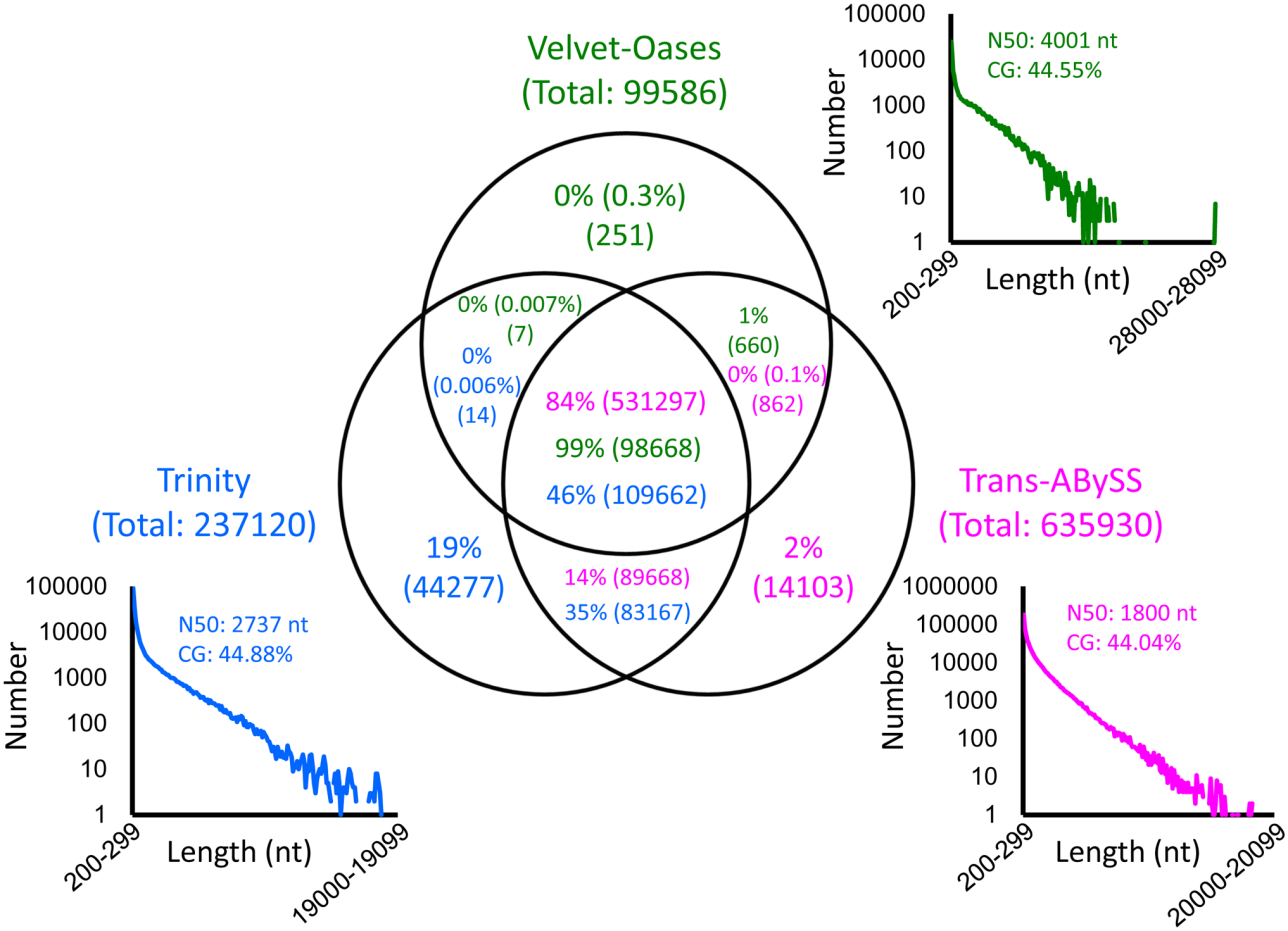
Comparisons between transcriptomes deduced by different assemblers. Scatter plot graphs show the length distribution of *IS*-transcripts deduced by Trinity (blue), Trans-ABySS (magenta) and Velvet-Oases (green). As suggested by the total number and N50 length of *IS*-transcripts, Velvet-Oases tended to give long *IS*-transcripts at the expense of their total number, while Trans-ABySS deduces a large number of short *IS*-transcripts. Trinity was intermediate with respect to these parameters. The Venn diagram shows how much one assembler covers the *IS*-transcripts deduced by the other assemblers. The number of *IS*-transcripts is given in parentheses. Trinity covered almost all the *IS*-transcripts deduced by either Velvet-Oases or Trans-ABySS.

There is no consensus in terms of the best *de novo* assemblers. So, we tested three widely used algorisms, Trinity [15], Trans-ABySS [16] and Velvet-Oases [18]. Each algorism produced a large number of *IS*-transcripts: Trinity, 237,120 [N50 (the length of an *IS*-transcript whose order is 50% of all *IS*-transcripts): 2,737 nt]; Trans-ABySS, 635,930 (N50: 1,800 nt); Velvet-Oases, 99,586 (N50: 4,001 nt) [Figure 2; note that these sets of *IS*-transcripts (transcriptomes) can be downloaded from our repository site ‘IMORI’ (http://antler.is.utsunomiya-u.ac.jp/imori/)]. These three transcriptomes were compared with each other by blast (NCBI; v2.2.27+x64-linux; E-value threshold: e-5), revealing that Trinity covered almost all *IS*-transcripts in Trans-ABySS (∼98%) and Velvet-Oases (∼99%) (Figure 2). Thus, the Trinity data set seemed to be more comprehensive. So, here we applied the following analyses to this Trinity-deduced transcriptome.

### Trinity-deduced transcriptome in this study considerably covers transcript information in newts

**Figure 3:**
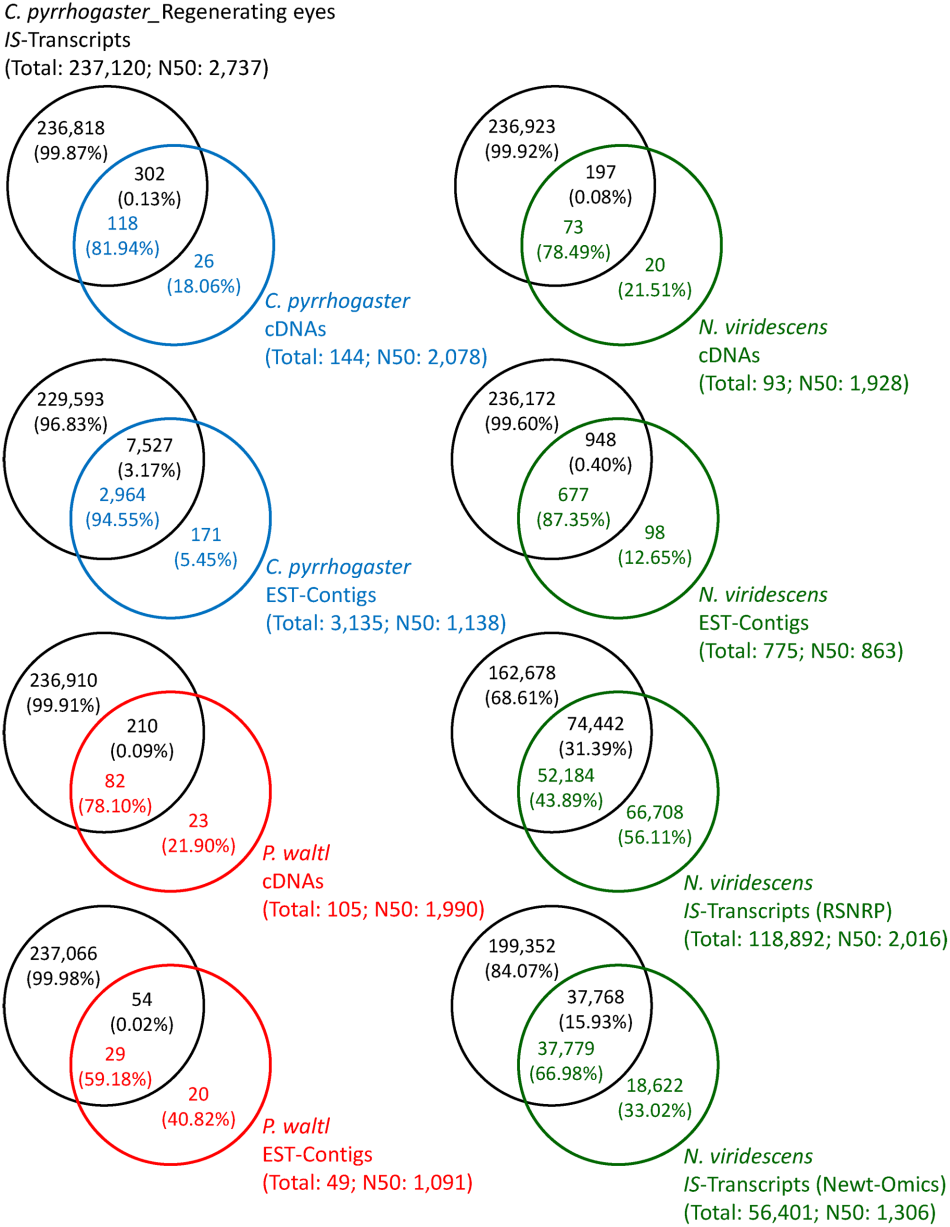
Comparisons with other newt transcriptomes. Black circles: *IS*-transcripts of this study. Blue circles: cDNAs and EST-contigs reported in *C. pyrrhogaster*. Red circles: cDNAs and EST-contigs reported in *P. waltl*. Green circles: cDNAs, EST-contigs and *IS*-transcripts reported in *N. viridescens*. The values in each circle (written in corresponding color) mean the number and ratio of *IS*-transcripts, cDNAs or EST-contigs.

To validate the current Trinity-deduced transcriptome, we first investigated what extent it covers transcript information in newts. For this, we compared this transcriptome, by blastn (E-value threshold: e-5), with other transcriptomes (cDNAs, ESTs, and *IS*-transcripts) in three species (*C. pyrrhogaster*, *Pleurodeles waltl*, *Notophthalmus viridescence*), which were collected from databases in DDBJ/NCBI and from sequence repository sites for *N. viridescence*: Red Spotted Newt Resource Page (RSNRP) http://sandberg.cmb.ki.se/redspottednewt/ [5] and Newt-Omics http://newt-omics.mpi-bn.mpg.de/ [6] (Figure 3). ESTs were assembled into contigs by the CAP3 program [22], before comparison. This analysis revealed that the current transcriptome in early regenerating eyes (day 0–14 po) contains >78% information of cDNAs/ESTs (i.e., mRNA) reported in these three species so far, except for 59% of ESTs in *P. waltl*; notably, in *C. pyrrhogaster* ∼82% of cDNAs and ∼95% of EST-contigs were matched (Figure 3). In addition, the current transcriptome covered ∼44% and ∼67% of the *IS*-transcripts for *N. viridescence* (RSNRP and Newt-Omics, respectively). On the other hand, these *N. viridescence* data covered only ∼31% and ∼16% of the current transcriptome, respectively. Thus, the current transcriptome comprehensively covers transcript information in *C. pyrrhogaster*.

### Characterization of the current transcriptome

#### Functional annotation

**Figure 4:**
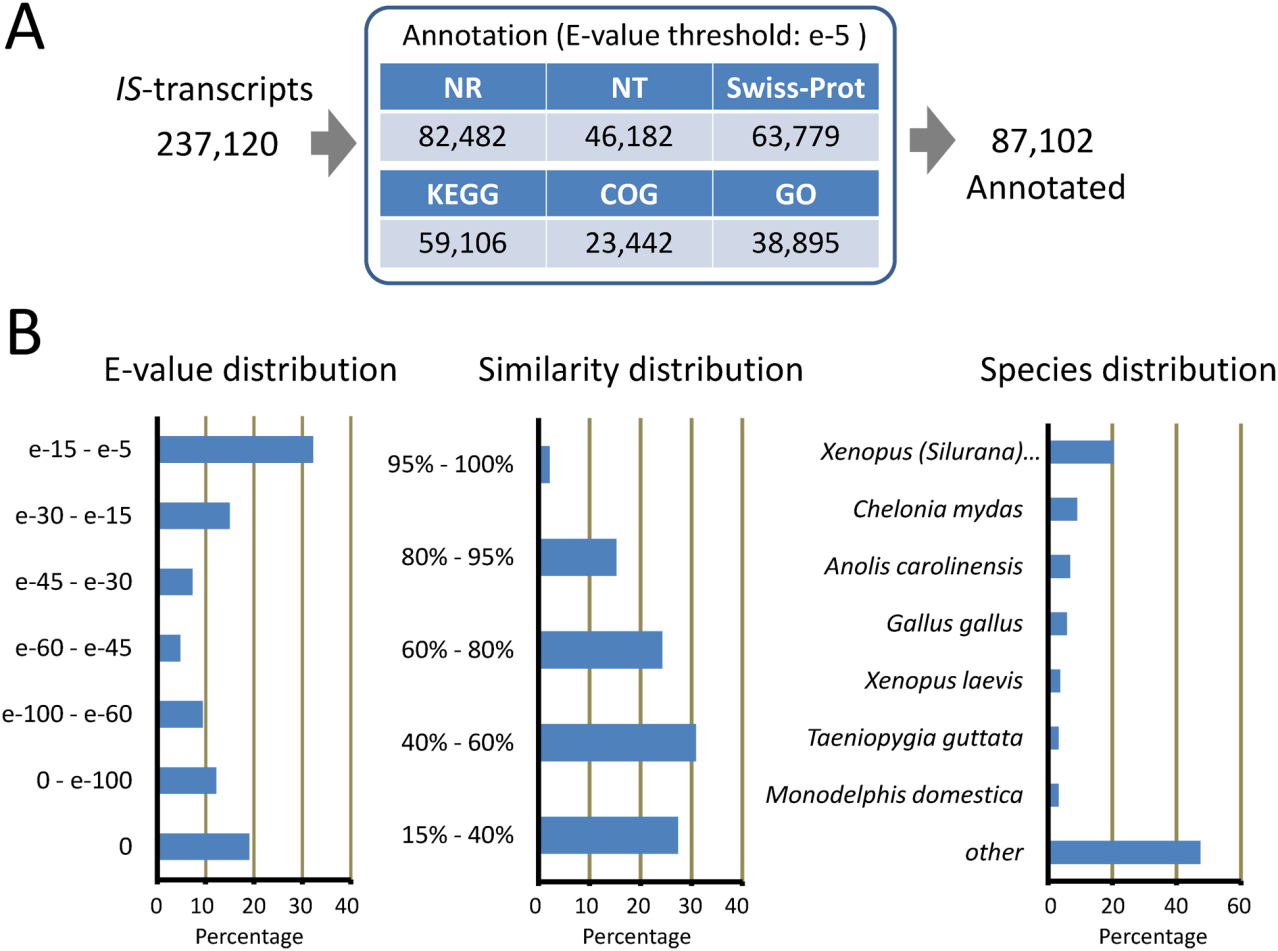
Functional annotation. **A.** Summary of annotation. **B.** E-value-, similarity (% identity)- and species-distribution in NR annotation. Species-distribution indicates that many of the *C. pyrrhogaster IS*-transcripts are close to genes of amniotes as well as those of amphibians such as *Xenopus (Silurana)* and *Xenopus laevis*, rather than fishes (e.g., *Danio rerio*, 2.9%; *Oryzias latipes*, 1.9%; these are included in ‘*other*’). Interestingly, within amphibians, the newt seems to adhere to *X. (Silurana)* rather than to *X. laevis*.

To further characterize the current transcriptome (237,120 *IS*-transcripts), physiological functions of each *IS*-transcript were predicted by annotation, which carried out by blast with NR, NT, Swiss-Prot, KEGG, COG and GO databases. Finally, a total of 87,102 *IS*-transcripts (range: 201–19,064 nt; N50: 1,202 nt) were annotated (Figure 4A; for details, see Table S2). For example, in NR annotation, 82,482 *IS*-transcripts (94.7% of all annotated transcripts) were enriched; 67.7% of them had an E-value of <e-5, and 41.8% had a similarity of >60%, indicating that many of the NR-annotated *IS*-transcripts contain the sequence information of genes highly homologues to those found in NR database (Figure 4B); species distribution seemed to reflect the position of this animal in phylogeny (Figure 4B). 38,895 of the NR-annotated *IS*-transcripts were also dealt GO terms (Figure 4A). As shown in GO classification (Table 1) as well as in COG- and KEGG-classification (Table S3 and S4), these *IS*-transcripts were assigned to various functional categories and pathways. On the other hand, the remaining 150,018 *IS*-transcripts (range: 201–11,726 nt; N50: 290 nt) were not annotated.

**Table 1:** GO classification.

Biological process	
Class	Count
cellular process	29,570
single-organism process	25,093
metabolic process	21,542
biological regulation	19,602
regulation of biological process	18,451
response to stimulus	14,838
multicellular organismal process	13,061
developmental process	11,328
signaling	11,013
cellular component organization or biogenesis	10,934
localization	10,576
positive regulation of biological process	8,574
establishment of localization	8,345
negative regulation of biological process	7,416
immune system process	3,363
locomotion	3,320
reproduction	2,907
reproductive process	2,768
biological adhesion	2,223
multi-organism process	2,222
growth	1,992
rhythmic process	442
cell killing	97

Cellular component	
Class	Count
cell	29,452
cell part	29,452
organelle	21,715
membrane	15,252
organelle part	13,910
membrane part	9,970
macromolecular complex	8,397
membrane-enclosed lumen	6,813
extracellular region	2,349
cell junction	1,989
synapse	1,713
extracellular region part	1,520
synapse part	1,205
extracellular matrix	894
extracellular matrix part	336
nucleoid	72

Molecular function	
Class	Count
binding	26,423
catalytic activity	13,617
transporter activity	2,690
molecular transducer activity	2,323
enzyme regulator activity	2,160
receptor activity	2,005
nucleic acid binding transcription factor activity	1,913
protein binding transcription factor activity	1,264
structural molecule activity	1,085
electron carrier activity	223
channel regulator activity	207
antioxidant activity	90
chemorepellent activity	66
receptor regulator activity	58
translation regulator activity	50
chemoattractant activity	30
metallochaperone activity	10
morphogen activity	3
nutrient reservoir activity	3
protein tag	1

#### Inferred proteome

**Figure 5:**
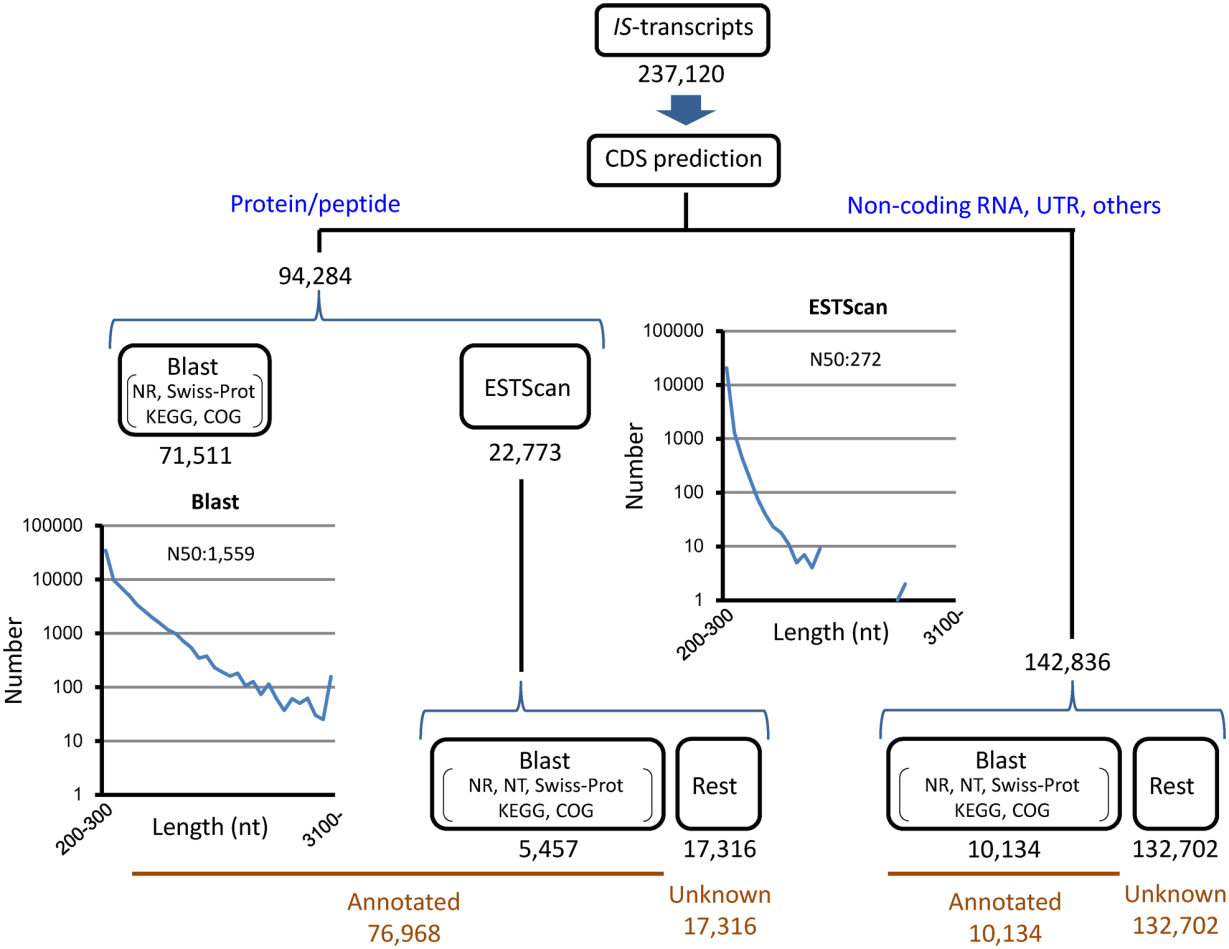
CDS prediction. Blastx predicted CDSs in 71,511 *IS*-transcripts (for the length distribution, see the graph ‘Blast’; for protein/peptide sequences, see Table S5) while ESTscan predicted CDSs in 22,773 *IS*-transcripts (for the length distribution, see the graph ‘ESTScan’; for protein/peptide sequences, see Table S6).

We carried out CDS and protein/peptide prediction independently of functional annotation (Figure 5). Protein/peptide sequences were first predicted in 71,511 of 237,120 *IS*-transcripts from the top hit sequence in blastx with NR, Swiss-Prot, KEGG, and COG databases (range: 20–6,124 aa; N50: 211 aa; Table S5), and then in 22,773 of the remnant *IS*-transcripts by ESTScan (range: 29–2,448 aa; N50: 90 aa; Table S6). Finally, these data were combined to the above annotation results. By this procedure, most of the predicted protein/peptides (76,968/94,284) were annotated; 5,457 ESTScan-predicted protein/peptides (range: 31–2448 aa; N50: 107 aa), of which *IS*-transcripts had a range of 201–11,070 nt (N50: 327 nt), were mostly unknown, uncharacterized, unnamed or hypothetical proteins. On the other hand, 142,836 *IS*-transcripts were unlikely to encode protein/peptides; 10,134 (range: 201–15,043 nt; N50: 454 nt) of them were annotated, mostly showing similarity to non-coding RNA and genomic sequences including the untranslated region (UTR) in other species.

17,316 *IS*-transcripts (range: 201–7,076 nt; N50: 262 nt) whose CDSs were predicted by ESTScan (Table S7) and the remaining 132,702 transcripts (range: 201–11,726 nt; N50: 290 nt) were not annotated (‘Rest’ in Figure 5). These were mostly short (∼80% of the former and ∼70% of the latter were 200–400 nt), but interestingly identical sequences [149 (range: 68–1915 aa; N50: 123 aa) of the former and 996 (range: 202–9052 nt; N50: 790 nt) of the latter] were also found in the newt limb blastema (R. M. Casco-Robles, unpublished data). Therefore, all of them cannot be associated with errors in *de novo* assembly, as revealed in *N. viridescence* [6].

In future studies, we need confirm the presence of these molecules as well as their physiological functions in this species.

### qPCR analysis of gene expression which has been inferred in early processes of retinal regeneration demonstrates the applicability of the current transcriptomes

**Figure 6:**
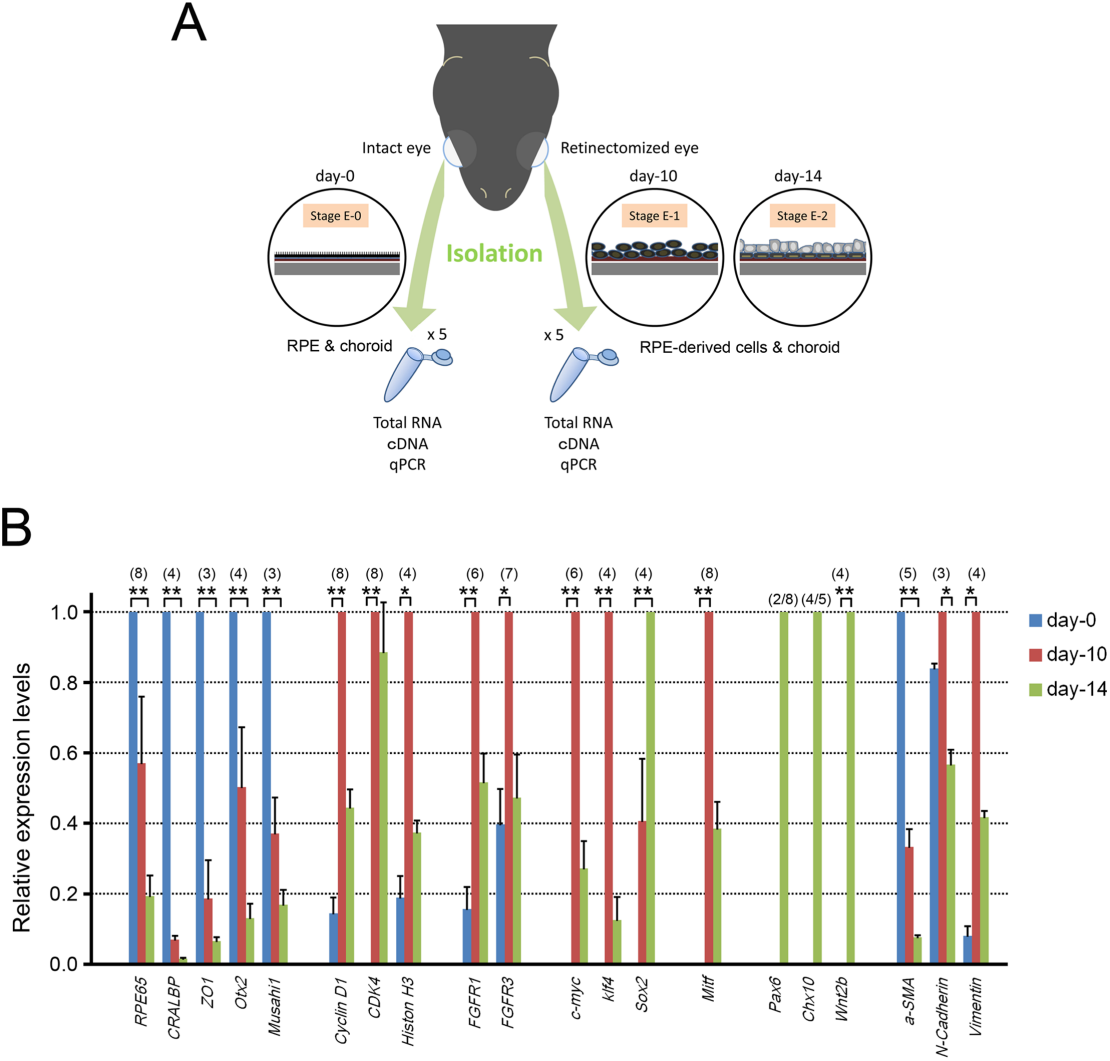
Validation by qPCR. **A.** Workflow of sample preparations for qPCR. RPE-choroid tissues harvested from the right eyes (intact eye) of 5 retinectomized animals at day-10 or day-14 were used for the normal RPE, i.e., the day-0 (Stage E-0) sample. RPE-derived cells harvested together with the choroid from the left eyes (retinectomized eye) of the same animals were used for the day-10 (Stage E-1) or day-14 (Stage E-2) sample. 3 different day samples (day-0, -10 and -14) were randomly grouped as one set of samples for qPCR. For more details, see Methods. **B.** qPCR analysis. 20 selected genes which have been suggested or inferred to be regulated in an early phase of retinal regeneration were found in the current *in silico* transcriptomes. Changes in their relative expression level until day-14 po were examined. Results represent means and SE. The number in parenthesis indicates the number of independent sample sets (see Methods) except for *Pax6* and *Chx10*, whose expression was detected in 2 of 8 sample sets and 4 of 5 sample sets, respectively at day-14 po only. Statistical significance based on Sheffe’s test following the Friedman test (*: *p*<0.05; **: *p*<0.01), except for *Pax6* and *Chx10*.

The current transcriptomes contained much information of genes which have not been identified in *C. pyrrhogaster* but whose transcription has been hypothesized to be regulated in early processes of retinal regeneration. Here, to validate the applicability of the current transcriptomes, we carried out qPCR analysis of selected 20 genes (Table S1) with cDNA samples prepared from RPE-choroid tissues or RPE-derived cells-choroid tissues which were harvested from intact (day-0) or retinectomized (day-10 or day-14) eyes respectively (Figure 6A); for unidentified genes (*CRALBP/RLBP1*, *ZO1*, *Cyclin D1*, *CDK4*, *Histone H3*, *c-Myc*, *Klf4*, *N-Cadherin*, *α-SMA*, *Vimentin*, *Chx10*/*Vsx2*, *Mitf*, *FGFR1*, *FGFR3*), the sequence of the target transcript was confirmed by conventional molecular cloning and Sanger DNA sequencing using the same tissue samples as those used for qPCR.

The results (Figure 6B) revealed that the expression levels of many genes characterizing RPE cells (*RPE65*, *CRALBP/RLBP1*, *ZO1*, *Otx2*, *Musashi1a/c*) gradually decreased upon retinectomy, while the expression of genes for the cell-cycle (*Cyclin D1*, *CDK4*, *Histone H3*) and growth signaling (*FGFR1*, *FGFR3*) was up-regulated to reach a maximum level by day-10 po when almost all RPE cells had entered the cell cycle (Stage E-1). Intriguingly, pluripotency factors except for *Oct4* (i.e., *c-Myc*, *Klf4*, *Sox2*) were expressed between day-0 and day-10 po; *Oct4* was not found in the current transcriptomes. Up-regulation of these three factors and lack of *Oct4* expression were also reported in early lens and limb regeneration of *N. viridescence* [23]. A microphthalmia factor *Mitf*, a marker gene for immature or uncommitted RPE cells [24], was also expressed by day-10 po but then its expression level decreased. On the other hand, marker genes for retinal stem/progenitor cells (*Pax6*, *Chx10/Vsx2*) were expressed at day-14 po when two rudiment layers (pro-NR and pro-RPE) for the prospective neural retina and RPE just appear (Stage-E2). Interestingly, the expression of *Wnt2b* was coincidental. Wnt/β-catenin signaling has been suggested to promote differentiation of the RPE while protecting cell-fate switching of the uncommitted RPE into the neural retina in embryonic eye development [11], [24].

In *PVR* leading to the loss of vision after retinal injury in humans, RPE cells transform into mesenchymal cells such as myofibroblasts, probably by passing through a stem-cell state [9]. In the newt, expression of mesenchymal markers (*N-Cadherin*, *Vimentin*) seemed to be up-regulated by day-10 po and then decreased while the relative expression level of a marker of myofibroblasts, *α-SMA* (smooth muscle actin), obviously decreased (Figure 6B).

Consequently, the current transcriptomes could be a good tool to identify or screen candidate genes which might be involved in early processes of retinal regeneration.

### Implications for reprogramming of adult newt RPE cells into a multipotent state

In the adult newt, the RPE is a sole cell source for retinal regeneration in the posterior eye [8]. Upon retinectomy, RPE cells are detached from each other and leave the basement membrane, forming cell aggregates, while entering the S-phase of cell-cycle (Stage E-1). This event typically occurs between day-5 and day-10 po. The RPE-derived cells at Stage E-1 form the pro-NR and pro-RPE layers by day-14 po (Stage E-2).

In the previous studies [25], [26], we demonstrated that an RNA-binding protein Musashi-1, whose expression is restricted in the nucleus of the intact RPE cells, changes its location into the cytoplasm upon retinectomy, although the amount of the transcripts tends to be decreased as suggested in the current qPCR. This pattern of Musashi-1 expression was observed in almost all of the RPE-derived cells at Stage E-1 uniformly. As the regeneration proceeds to Stage E-2, Musashi-1 expression was down-regulated along the pro-RPE layer, while sustained along the pro-NR layer [26]. Since the cytoplasmic expression of Musashi-1 is a character of neural stem/progenitor cells [27], these observations led us to an implication that the RPE cells are reprogrammed into a multipotent state of cells by Stage E-1, and specified into 2 cell populations forming the pro-NR and pro-RPE layers between Stage E-1 and E-2. However, the nature of the RPE-derived cells remains unclear.

The current qPCR revealed that gene expression suggesting multipotent properties of cells is up-regulated upon retinectomy, while gene expression for original RPE characters is down-regulated, giving us an insight for the cell reprogramming. Interestingly, certain pluripotency factors (*c-Myc*, *Klf4*, *Sox2*) as well as *Mitf* were first detected in day-10 samples (Stage E-1). On the other hand, *Pax6* was detected only in day-14 samples. In the previous study, we detected *Pax6* expression at day-10 po, being earlier than *Chx10-1*/*Vsx1* (a retinal progenitor marker) at day-14 po [28]. This inconsistency may be due to the difference in the template for PCR: in the previous study, we used a PCR-amplified library of cDNAs as the template, and therefore the detection sensitivity should have been higher. Taken together, our results suggest that the intact adult newt RPE cells are not comparable to either immature/uncommitted state of RPE cells or retinal stem/progenitor cells, as well as a possibility of reprogramming of RPE cells into such multipotent cells upon retinectomy. On the next stage of this study, we must carry out immunohistochemistry and single-cell qPCR to address the expression of these factors in the RPE-derived cells at Stage E-1 (our study on this subject was published [29] while the current article was under review).

The current qPCR with day-14 samples detected a factor (*Chx10*/*Vsx2*) suggesting the presence of retinal progenitor cells and that (*Wnt2b*) inferred to be involved in the RPE differentiation. *Sox2*, which is also a marker of retinal stem/progenitor cells [30], increased in its expression level between day-10 and day-14, whereas *c-Myc*, *Klf4* and *Mitf* were declined. These expression patterns seem to support our previous implication that the RPE-derived cells are specified into two cell populations from which the pro-NR and pro-RPE layers are formed, leading a possibility that the cells in the pro-NR and pro-RPE layers might be correspond to the retinal progenitor cells and RPE cells which just started their differentiation, respectively. In addition, the expression of *α-SMA*, which is observed in the choroid tissues (data not shown), was obviously declined to a minimal level at day-14, revealing a contrast to human *PVR* in which the RPE-derived multipotent cells transform into myofibroblasts. Thus, in the adult newt system, the RPE-derived cells (possibly in a multipotent state) direct properly and seem to generate retinal progenitor cells for regeneration of a missing neural retina.

In the current study, we did not carry out further analyses because the purpose of the study was to establish a good transcriptome database. However, our study would move onto the next stage at histological and functional levels.

### Conclusions

To facilitate investigations of adult newt retinal regeneration, we provided *in silico* transcriptomes covering information of genes which are expressed in eyeballs containing Stage E-0 to E-2 regenerating retinas. Gene expression patterns revealed by qPCR demonstrate the usefulness of the transcriptomes for the study of early processes of retinal regeneration. This tool can be applied to the next comprehensive gene screening steps to uncover essential mechanisms underlying reprogramming, cell-cycle re-entry/proliferation, and patterning in adult newt retinal regeneration.

## Supporting Information

Table S1 **Primers and probes for PCR targets.**
(XLSX)

Table S2 **Summary of annotation.**
(XLSX)

Table S3 **COG classification.**
(XLSX)

Table S4 **KEGG classification.**
(XLSX)

Table S5 **Proteins/peptides predicted by blast.**
(XLSX)

Table S6 **Proteins/peptides predicted by ESTScan.**
(XLSX)

Table S7 **Unknown proteins/peptides predicted by ESTScan.**
(XLSX)
